# Characterisation of PAHs in Outdoor Air Pollution at Schools in a Medium-Sized Town, Hungary

**DOI:** 10.3390/toxics14040326

**Published:** 2026-04-15

**Authors:** Bettina Eck-Varanka, Nóra Kováts, Attila Szűcs, Katalin Hubai

**Affiliations:** 1 Centre for Natural Sciences, University of Pannonia, Egyetem Str. 10, 8200 Veszprém, Hungary; eck-varanka.bettina@mk.uni-pannon.hu (B.E.-V.); hubai.katalin@mk.uni-pannon.hu (K.H.); 2 Sustainability Solutions Research Laboratory, University of Pannonia, Egyetem Str. 10, 8200 Veszprém, Hungary; szucs.attila@htk.uni-pannon.hu

**Keywords:** resuspended dust, ecotoxicity, PAHs, source apportionment, ILCR

## Abstract

Atmospheric particulate matter poses a high risk by carrying potentially toxic components such as polycyclic aromatic hydrocarbons (PAHs). The major sources of these potentially toxic compounds include traffic-related emissions and winter heating, implying the combustion of fossil fuels or biomass. Air pollution, especially chronic exposure, poses the most serious human health hazard in childhood, and several studies emphasise the importance of research on the potential impacts of air pollution in school environments. While indoor air quality studies are already available in Hungary, investigations on outdoor air pollution in school environments are missing. To fill this gap, in a medium-sized Hungarian town, Veszprém, six schools were selected to assess air quality in the outdoor environments where schoolchildren spend their breaks and have physical training. These schools represent different locations and conditions, from high-trafficked sites to suburban environments. Using resuspended dust samples, environmental quality was assessed based on PAH contents of the samples and ecotoxicity tests (*Vibrio fischeri* bacterial bioassay). Ecotoxicity of the samples moved in a wide range, from highly toxic to non-toxic. PAH measurements indicated considerable contamination in the case of one sample taken from a suburban area. Source apportionment demonstrated that winter heating is also an important pollution source.

## 1. Introduction

Atmospheric particulate matter (PM) poses an increasing risk to both human and environmental health, according to the EU directive on air quality (2008/50/CE). Specific target values were set for PM10 and PM2.5 [1]. Polycyclic aromatic hydrocarbons (PAHs) occur both in the gas phase and adsorbed on atmospheric particles (particulate phase) [2]. PAHs originate from the incomplete combustion of fossil fuels, with diesel-powered vehicles being an important source. Besides well-known toxic properties, several PAHs show carcinogenic properties. The International Agency for Research on Cancer (IARC) reviewed experimental data for 60 individual PAHs and set three groups: carcinogenic to humans (group 1), probably carcinogenic to humans (group 2A), and possibly carcinogenic to humans (group 2B) [3]. In 2016, the IARC even classified particulate matter and outdoor air pollution as carcinogenic to humans (group 1) [4].

The main natural sinks and reservoirs of PAHs are air and dust [5]. PAHs settle in dust through dry and wet deposition, so they can be taken up by people via dermal contact, inhalation and dust absorption, which are the most common exposure routes of PAHs [6]. The chemical composition of PM depends on the emission sources; source identification of PM compounds is necessary to improve strategies for effective air quality management [7]. Traditionally established PAH isomer ratios have been in use to allocate PAH emission sources in any environmental sample [8]. Based on PAH concentrations, the incremental lifetime cancer risk (ILCR) can be calculated, which can provide insight into the health risks faced by students [9].

Chronic air pollution exposure poses the most serious health hazard in childhood [10]. It can cause cardiovascular and pulmonary symptoms, damage neurodevelopmental skills in children [11] or even reduce the level of cognitive functions [12]. Many PAHs are known to increase the risk of cancer [3].

Since the early 2000s, national and international projects have focused on assessing indoor air quality in primary schools and examining the impact of indoor air pollutants on children’s health, in order to develop guidelines for creating a healthy school environment (AIRMEX; SEARCH; SINPHONIE; InAirQ) [13]. The studies have mostly been limited to indoor air quality (Volatile Organic Compounds (VOCs), PM10, CO, CO_2_, NO_2_, O_3_); however, the InAirQ project has shown that indoor sources contribute less to PM2.5 mass concentrations, confirming the fact that fine particles are largely of outdoor origin [14].

In Hungary, several schools are located near high-traffic roads. These children can be exposed to air pollution during outdoor activities; in addition, outdoor air quality influences indoor air quality as well [15]. A survey in Hungary asked 9–11-year-old children about the health impact of harmful environmental factors. The results of the study show that they are aware of the risks around them and their opinions are largely influenced by their individual experiences, but they do not always see the connection between an environmental factor and a disease [16].

One major tool to estimate the ecotoxicological character of outdoor air quality is the bioluminescent bacterial test. In our previous work, a specific direct contact ‘whole aerosol test’ was developed by our team, which allows direct contact between *Vibrio fischeri* test bacteria and PM-bound contaminants [17]. As such, environmentally relevant exposure pathways can be mimicked. The direct contact test has been applied to characterise traffic-related emissions. In the case of emissions of diesel-powered vehicles, the test proved a good indicator of PAH-related ecotoxicity [18]. The bioluminescence inhibition bacterial bioassay could also respond to heavy-metal-polluted road dust samples [19,20]. The test in general is recommended as an efficient tool for the quantification of PM-associated biotoxic effects [21].

The specific aims of this study were (1) to provide a snapshot assessment of the outdoor air quality of these schools, based on PAH analysis and ecotoxicological screening, and (2) to identify the main pollution sources from the PAH ratios of target PAH levels.

## 2. Materials and Methods

### 2.1. Sampling

**Figure 1:**
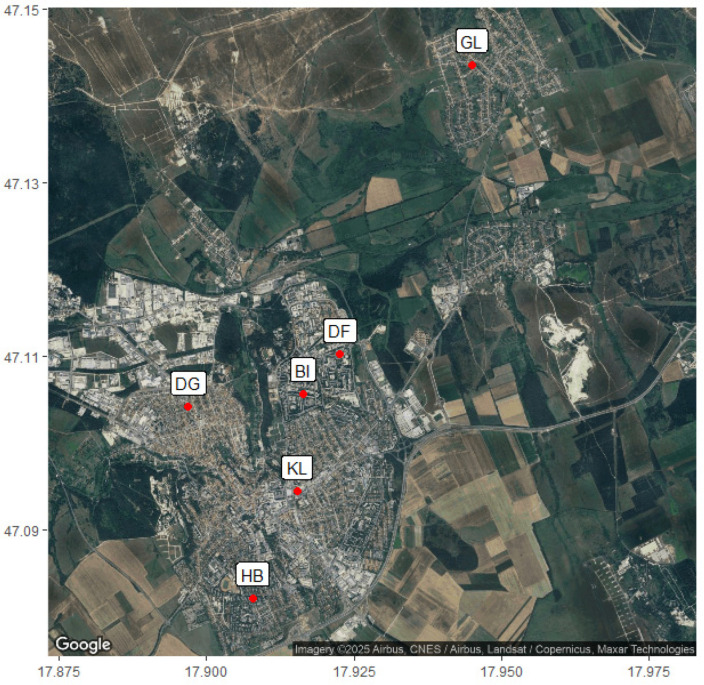
The location of the 6 schools on the map of Veszprém, created in RStudio (version 2024.12.1 Build 563) from a Google Maps satellite view image [22].

The sampling site was in a Hungarian county seat, Veszprém, which has more than 55,000 inhabitants. The elementary schools were chosen based on parameters affecting air pollution, like location, proximity to roads, or traffic. A summary of the selected schools is given in Table 1. The location of the schools in Veszprém is shown in Figure 1.

**Figure 2:**
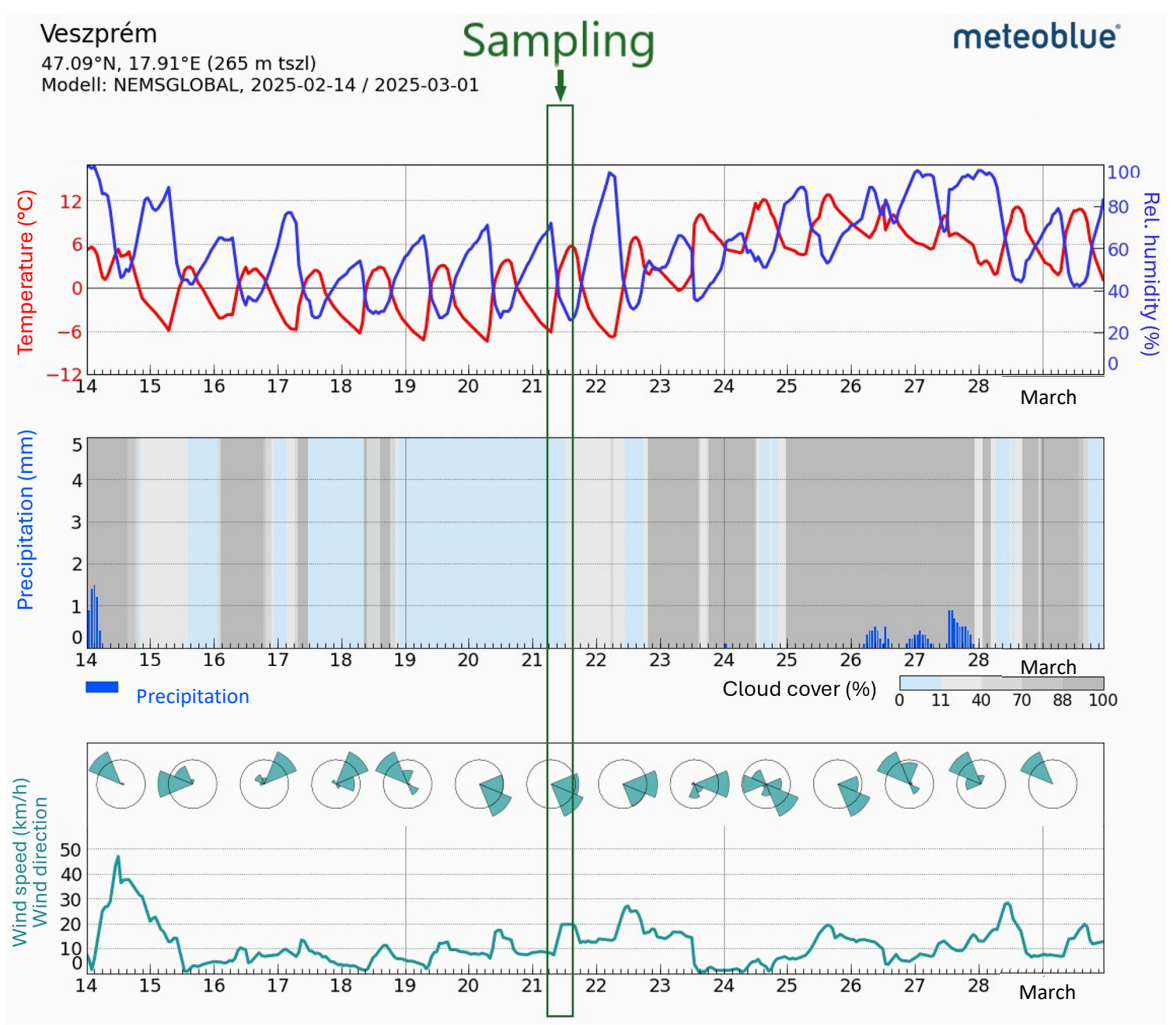
Typical weather conditions.

The date of the sampling was 21 February at temperatures between −3 and +3 °C. The sampling day was selected to represent (1) typical winter heating conditions (in Hungary, the official heating season stretches from 15 September to 15 May) and (2) a working day in school. Weather conditions are represented in Figure 2.

Resuspended dust samples were collected by manual sweeping with a natural brush (pig bristle) from the fence bastions of schools. Fence bastions were used as they are closer to the breathing zone of children; also, these are the surfaces where direct dermal contact can occur. Moreover, children are more likely to ingest settled dust through hand-to-mouth contact [23]. At each sampling site, three dust samples were collected and were evenly mixed, providing a composite dust sample [24]. Samples were stored in glass vials to avoid adhesion of PAH compounds. Approximately 4 g of dust was collected from each sampling site. In the laboratory, the samples were sieved first through a 1 mm mesh to remove grit and organic materials, then a 0.1 mm mesh sieve was used, and the samples were stored in a refrigerator until further analysis.

### 2.2. Analytical Measurements

Polycyclic aromatic hydrocarbon concentrations were measured by gas chromatographic mass spectrometry (Agilent 6890GC 5973E MSD GC-MS, Agilent Technologies, Santa Clara, CA, USA) in the laboratory of the ELGOSCAR-2000 Environmental Technology and Water Management Ltd. according to ISO/IEC 17025:2018 standard [25]. The GC-MS measurements were made from the 1 mm fraction of dust. Beside the USEPA 16 priority PAHs (ΣPAHs) (Naphthalene—NP, Acenaphthylene—ACY, Acenaphthene—ACE, Fluorene—FL, Phenanthrene—PHE, Anthracene—ANT, Fluoranthene—FLA, Pyrene—PYR, Benzanthracene—BaA, Chrysene—CHR, Benzo(b)fluoranthene—BbF, Benzo(k)fluoranthene—BkF, Benzo(a)pyrene—BaP, Bibenzo(a,h)anthracene—DahA, Indeno1,2,3CD-pyrene—IcdP, Benzo(g,h,i)perylene—BghiP) 1-methyl-naphtalene (1MNP), 2-methyl-naphtalene (2MNP) and Benzo(e)pyrene (BeP) were also measured.

### 2.3. Source Identification

Low molecular weight (LMW) PAHs are generally formed during low-temperature processes such as wood burning, while high-temperature processes (i.e., fuel combustion in engines) produce higher molecular weight (HMW) PAHs [26]. In the present study, PAH isomer ratios as LMW/HMW [8], FL/(FL + PYR) [27], FLA/(FLA + PYR), IcdP/(IcdP + BghiP) [28], BaP/(BaP + BeP) [29], BaP/BghiP [30], BaA/(BaA + CHR), and CHR/(CHR + BaP) [31] were used to predict the origins of PAHs.

### 2.4. Incremental Lifetime Cancer Risk (ILCR)

From the concentrations of individual PAHs, BAP (Benzo(a)pyrene) toxic equivalency (BAP-TEQ) was calculated using the following formula:

$$\sum BAP-TEQ=\sum \left(C_{i}\times {TEF}_{i}\right)$$

where Ci is the concentration of an individual PAH (μg/g), and TEFi is the toxic equivalency factor of the carcinogenic PAHs, that is 0.001 for NAP, ACE, ACY, FLA, PHE, FLU and PYR; 0.01 for ANT, CHR, and BghiP; 0.1 for BaA, BbF, BkF and IcdP; and 1 for BaP and DahA [32,33].

The ILCR values were calculated for the three exposure pathways by the following formulas [34]:

$$ILCRing=\frac{CS\times \left(CSFing\times \sqrt[{3}]{\frac{BW}{70}}\right)\times IRing\times EF\times ED}{BW\times AT\times 10^{6}}$$

$$ILCRinh=\frac{CS\times \left(CSFinh\times \sqrt[{3}]{\frac{BW}{70}}\right)\times IRinh\times EF\times ED}{BW\times AT\times PEF}$$

$$ILCRderm=\frac{CS\times \left(CSFderm\times \sqrt[{3}]{\frac{BW}{70}}\right)\times SA\times AF\times ABS\times EF\times ED}{BW\times AT\times 10^{6}}$$

where CS is the sum of converted concentrations of individual PAHs (μg/g) based on the TEQ (BAP-TEQ). The total health risk is the sum of risks from the three exposure pathways. The value and reference of each parameter are listed in Table 2.

### 2.5. Toxicity Assessment

Ecotoxicological testing was performed as described in ISO 21338:2010: Water quality—Kinetic determination of the inhibitory effects of sediment, other solids and coloured samples on the light emission of *Vibrio fischeri*/kinetic luminescent bacteria test [39]. For this test, a suspension was prepared from the 1 mm and 0.1 mm fractions of the dust samples with 100 mg dust + 1 mL of 2% NaCl solution.

## 3. Results and Discussion

These results are based on a snapshot assessment, conducted on a winter day, which represents typical heating and weekday conditions. However, such snapshot assessments of street/road dust can be widely found in the relevant literature, even providing input data for ILCR calculations [40]. A similar sampling strategy was reported by Rybak et al. [41], where road dust sampling was followed by chemical assessment and mutagenicity testing by the SOS Chromotest. Jancsek-Turóczi et al. [42] state that ‘dust particles preserve cumulative signatures of particles’ in a work where a specific, automated resuspended dust sampling device was introduced.

### 3.1. Levels and Distribution of PAHs in Resuspendable Dust

Altogether 19 PAHs were measured from the dust samples, including 16 USEPA priority pollutants. In all 6 schools, four-ring PAHs dominated; the distribution of other PAHs was quite diverse. Table 3 shows the measured PAHs concentrations in schools.

**Figure 3:**
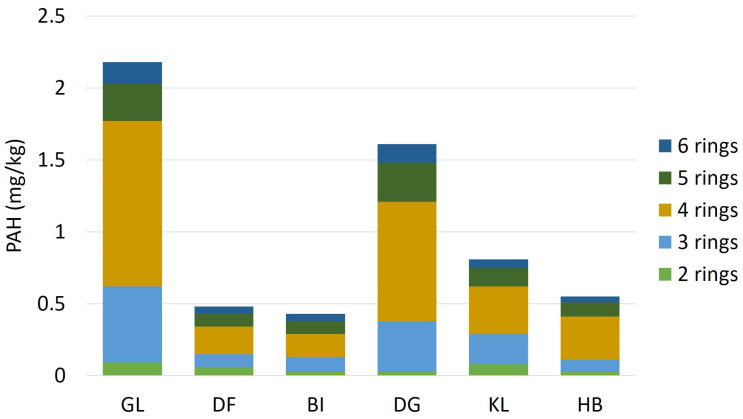
Distribution of PAH compounds according to ring number in the sampled schools.

The concentration of ∑PAHs ranged from 0.43 to 2.18 µg g^−1^. The distribution of PAH compounds between schools according to ring numbers is shown in Figure 3. Almost all individual PAHs and the ∑PAHs were highest at sampling sites GL and DG. Similar values were reported by Jancsek-Turóczi et al. [42]. When resuspended road dust samples were taken in the inner city of Veszprém, ∑PAHs were in the range of 1.46–3.48 µg g^−1^. Fluoranthene was detected in the highest concentration (0.07–0.63 µg g^−1^) while 1-methyl-naphthalene and acenaphthene were below the detection limit at every school. The results are comparable to other urban dust measurements from this European region. Škrbić et al. measured as high as 0.59 μg g^−1^ concentration of fluoranthene in winter road dust samples in Novi Sad (Serbia), a city even comparable in size [40]. Lorenzi et al. examined ΣPAHs concentrations in urban dust samples from England, which ranged from 0.56 to 46 µg g^−1^ with a significantly high maximum [43]. The PAH content of the samples was also measured by particle size, which showed that particles smaller than <63 µm and the 1000–2000 μm fraction had the highest PAH concentrations. Most authors emphasise that while relatively few data are available on PAH contamination of road dust, the health risk associated with road dust resuspension requires more thorough research [41,44].

Regarding the measured PAH concentrations, it is important to note that PAHs in ambient air are divided between the vapour and particulate phases based on the volatility of the compound, temperature, humidity, and the concentration of free radicals [45]. Low ambient air temperature can cause a higher amount of PAHs in the particulate phase. At all sampling sites, the HMW PAHs were present in a higher proportion (64.2–80%) than lighter ones. Oliveira et al. demonstrated a close correlation between outdoor and indoor air quality in urban schools of Portugal [29]. Based on the indoor/outdoor ratios, the LMW PAHs had higher indoor emission sources, while the 4–6 ring PAHs had mainly ambient sources [46]. The high HMW PAH ratios measured in our samples suggest that they are also significantly present indoors.

PAH profiles show clear spatial patterns. GL, although it belongs to Veszprém, is practically situated in a rural environment where detached houses are typical. These houses all have individual heating systems, commonly using gas, coal or wood. Emissions from winter heating can significantly affect air quality.

### 3.2. Source Identification

PAH ratios and double ratios were determined to predict the origins of PAHs. Table 4 shows the diagnostic ratios used in this study and the ranges for the evaluation. A commonly used PAH isomer ratio, ANT/(ANT + PHE), was not applied, as the concentration of anthracene was mostly below the detection limit.

**Figure 4:**
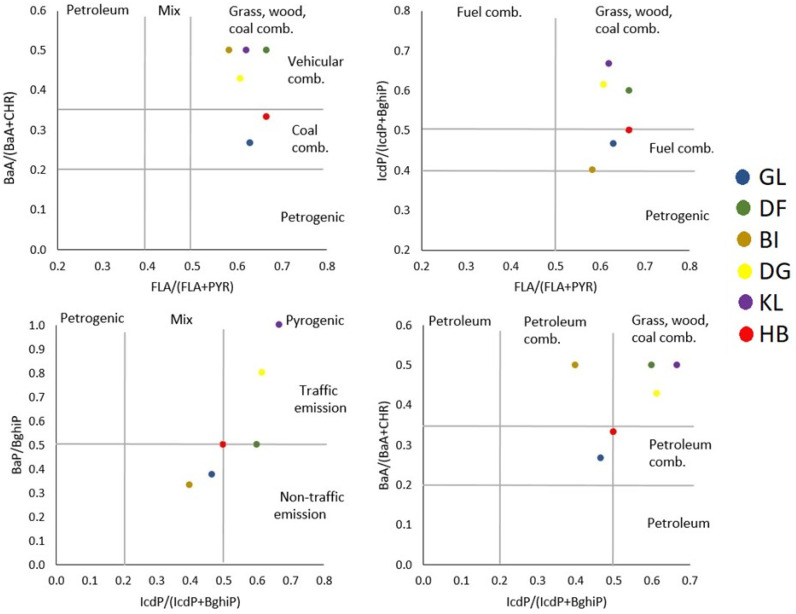
Cross plots for isomeric ratios in dust.

According to conservative diagnostic PAH ratios, the following assumptions can be made. The LMW/HMW ratio (<1) suggests the pyrogenic source in all six schools. FLA/(FLA + PYR) ratio indicates the contribution from biomass and coal combustion, as the values are above 0.5 in the case of all schools. When interpreting the IcdP/(IcdP + BghiP) ratio, some differences can be seen amongst schools, indicating that at sampling sites GL, BI, and HB, petroleum combustion plays a role as well. Contribution of coal combustion in GL and HB is suggested by the BaA/(BaA + CHR) ratio, being in the range of 0.2 and 0.35. The CHR/(CHR + BAP) ratio also supports this assumption, being higher than 0.76 in these sampling sites. Based on the BAP/BghiP ratio, the KL school appears in the upper right corner of Figure 4, showing the contribution of traffic-related emissions. This school is situated in the vicinity of the central bus station (distance is approximately 100 m, Table 1).

In a previous study [47], PAH distribution patterns accumulated by *Plantago* plants were investigated at different locations in the surroundings of Veszprém. The PAH sources observed very close to the KL school were the same as in the present study (coal combustion and vehicle emissions). As the ratio of BaP/(BaP + BeP) in this study ranges from 0.2 to 0.444, it suggests that the main cause of the pollution is rather the compounds formed during photolytic reactions than fresh particles. The separation of GL and HB from the other schools based on their PAH profile is clearly visible in the double-ratio diagrams in Figure 4.

Tobiszewski and Namieśnik [8] stress that diagnostic ratios in general give only indicative information on the share of different pollution sources, as the atmospheric presence of PAHs depends on various factors. The use of more than one diagnostic ratio is recommended [40]. However, individual ratios can be informative when compared to clearly identified pollution sources. For example, BaA/(BaA + CHR) and CHR/(CHR + BaP) ratios suggest a high share of residential coal combustion in GL and HB, which is also supported by the observation on heating patterns in these residential areas (please see Table 1).

### 3.3. Cancer Risk Assessment

To estimate the cancer risk of PAHs, guideline values for children (6–14 years) were used in calculations. BAP-TEQ values—which were calculated from the 16 priority pollutants of USEPA—and ILCR values from various exposure routes (ingestion, inhalation, dermal), as well as the total carcinogenic risk, can be found in Table 5.

The cancer risk from exposure routes was found to be in the following order: inhalation (10^−11^) < dermal (10^−6^) < ingestion (10^−6^). Ingestion of potentially contaminated dust or soil particles can be explained, for example, by eating dropped food, or hand-to-mouth or hand-to-object behaviours (reviewed by Gong et al. [23]. The daily average soil and dust ingestion rates for children are most likely around 50 mg day^−1^ [48].

According to the ILCR baseline, if the risk is under 10^−6^, then it is acceptable; between 10^−6^ and 10^−4^, there is potential risk; and if it is higher than 10^−4^, high risk can be anticipated [49]. Based on conservative models given in Table 2, in the case of GL, KL, and HB, the ILCR exceeded the criterion of 10^−6^, indicating potential health risk; the other three schools were also close to this limit. The results of a Polish study showed that PM_2.5_ and PM_2.5_-bound PAHs, both in outdoor and indoor air of kindergartens, are important sources of exposure of children to genotoxic substances [50]. The ILCR values in the present schools were higher than those measured for children in Iranian schools by Davoudi et al. [51] or in Polish urban dust measured by Rybak et al. [41], but significantly lower than in Pakistan [36].

The mechanistic interpretation of ILCR values, however, has serious limitations. These values, while indicating that atmospheric particles are significant carriers of airborne carcinogens [52], suggest that the potential cancer risk is associated with long-term exposure. In the case of contact with street dust, however, exposure might be significantly lower, which in turn means that risk seems to be overestimated. Actual exposure can also be reduced by, for example, weather conditions when children might prefer to stay indoors. Bearing in mind all limitations, some studies still suggest that overestimation can provide better protection of human health [53,54].

In our investigation, ILCR values might be used to define the most important exposure routes. Our results are in line with other studies conducted on resuspended dust. Generally, dermal contact is the main exposure route contributing to ILCR, followed by ingestion, while exposure via inhalation presents a low or negligible contribution [40]. As the risks from inhalation of dust (ILCR_Inhalation_) were about 10^5^ times lower compared to the risk from other exposure pathways, direct hazard to the respiratory system can be excluded.

### 3.4. Ecotoxicity

Ecotoxicity values showed great variability between samples and were very different for the two tested fractions (<1 mm and <0.1 mm). EC50 and EC20 values are given in Table 6.

Even though the GL sample had the highest amount of PAHs, it did not show any ecotoxic effects, similar to HB. The lowest EC50 value, however, indicating the strongest ecotoxic effect, was for the BI sample, which has the lowest PAHs level based on the analytical tests. The available literature is contradictory about possible relationships between the presence of PAHs and *Vibrio* ecotoxicity. Analysis of urban resuspended particulate matter (PM10) samples taken in Aveiro, a city in Portugal with a similar size to Veszprém, showed a close correlation between PAH content and EC50 values [55]. In indoor quality assessments, mostly HMW PAHs were found to contribute to *Vibrio* ecotoxicity [56], showing good agreement [57].

Although in the case of these indoor samples, the bioluminescent bacterium assay proved a reliable proxy for indicating human health risk, in the present study, no such sensitivity could be observed. Most likely, the presence of other potentially toxic compounds also contributed to the measured bioluminescence inhibition. Heavy metals have been shown to elucidate ecotoxic responses in urban samples [58]. It should be noted, however, that higher toxicity was recorded in the <0.1 mm fraction. Other studies also reported a negative correlation between particle size and ecotoxicity [59].

### 3.5. Comparison of Schools

Based on PAH profiles, GL and DG show the highest levels of PAH pollution. GL is situated in a suburban section of the city, while the location of DG is clearly rural. These are the areas where wood is dominating as the primary heating material. Wood burning is considered a sustainable energy source [60], and in Europe, the use of energy from renewable sources is promoted [61]. However, wood burning results in the emissions of a wide range of pollutants, including polycyclic aromatic hydrocarbons (PAHs) [62].

These schools seem to be at the highest risk, as outdoor air pollution levels have been found to have a determining effect on the indoor air quality of schools [63]. On the other hand, schools which are situated in neighbourhoods using district heating or gas (DF and BI) seem to be less exposed to air pollution, even in the inner parts of the city.

Though European studies suggest the high impact of traffic-related pollution on schoolchildren’s health [64], in the selected schools of Veszprém, biomass burning seems the dominant factor, except for KL, the school in the proximity of the central bus station. While the polluting effects of winter biomass burning are difficult to minimise, traffic-related air pollution can be reduced by, for example, the use of green walls [65].

## 4. Conclusions

This study investigated the source profile and health risks of PAHs in resuspended dust samples collected from school yards. In all sample sites, the proportion of four-ring PAHs was the highest, which can be attributed to vehicular emission, biomass and coal combustion sources. PAH pollution is clearly of pyrogenic origin; only the contribution ratio of biomass burning and traffic is different in schools. Based on PAH distribution patterns, spatial differences could also be found, showing high risk in areas with wood-based heating. A clear distinction can be found between schools situated in suburban/rural areas where wood-based heating is common and schools which are situated in areas where central heating or gas usage is dominant. Ecotoxicity of the <0.1 mm fraction of the samples, as measured by the *Vibrio fischeri* bioluminescence inhibition test, showed clear differences between schools, though ecotoxicity could not be associated with PAH levels. The BAP-TEQ values ranged from 0.0187 to 0.0925 mg kg^−1^, showing the presence of potentially carcinogenic compounds. Being the first Hungarian study conducted on outdoor air pollution in the neighbourhoods of schools, it highlights the need to consider road dust resuspension as an important factor affecting schoolchildren’s health. These results are also comparable to regional studies depicting the Central European situation.

## Figures and Tables

**Table 1:** Shortcuts of the schools and description of the neighbourhood.

School’s Name	Shortcut	Description
Gyulaffy László German Nationality Language Teaching Primary School	GL	Suburban/rural area, with detached houses. Main heating sources are wood, coal, and gas. The distance of the main road with high traffic is 300 m.
Deák Ferenc Primary School	DF	Housing estate with block houses. Main heating sources are district heating and gas. The distance of the main road with high traffic is 300 m.
Báthory István Sports School and Primary School in Veszprém	BI	Housing estate surrounded by block houses, the main heating source is district heating. The distance of the main road with high traffic is 900 m.
Dózsa György German Nationality Language Teaching Primary School	DG	Suburban, typically with detached houses. Main heating sources are wood, coal, and gas. The distance of the main road with high traffic is 1500 m.
Kossuth Lajos Elementary School in Veszprém	KL	Central, located in the city centre. The distance to the main bus station is 100 m, and the main road of the city runs in front of the school.
Hristo Botev German Nationality Language Teaching Primary School	HB	Suburban, typically with terraced houses, main heating sources are gas and wood to a lesser extent. The distance of the main road with high traffic is 500 m.

**Table 2:** Value and reference of parameters in health risk evaluation for PAHs.

	Abbrev.	Value	Unit	Ref.
Ingestion rate	IRing	200	mg day^−1^	USEPA 2011 [35]
Inhalation rate	IRinh	10	m^3^ day^−1^	Soltani et al., 2015 [36]
Dermal absorption fraction	ABS	0.13		USEPA 2011 [35]
Dermal adherence factor	AF	0.2	mg cm^2^	USEPA 2011 [35]
Dermal exposure area	SA	2800	cm^2^	USEPA 2011 [35]
Ingestion cancer slope factor	CSFing	7.3	mg kg^−1^ day^−1^	Peng et al., 2011 [37]
Inhalation cancer slope factor	CSFinh	3.85	mg kg^−1^ day^−1^	Peng et al., 2011 [37]
Dermal cancer slope factor	CSFderm	25	mg kg^−1^ day^−1^	Peng et al., 2011 [37]
Particulate emission factor	PEF	1.36 × 10^−9^	m^3^ kg^−1^	USEPA 2011 [35]
Body weight (6–14 year mean)	BW	34	kg	Hídvégi et al., 2024 [38]
Exposure frequency	EF	183	day year^−1^	Schooldays/year
Exposure duration	ED	8	year	
Averaging time	AT	2920	day	

**Table 3:** Measured PAHs concentrations in the 6 schools (µg PAH g^−1^ dust).

PAH		GL	DF	BI	DG	KL	HB
NP	Naphthalene	0.06	0.06	0.03	0.03	0.08	0.03
2MNP	2-methyl-naphthalene	0.03	0	0	0	0	0
1MNP	1-methyl-naphthalene	0	0	0	0	0	0
∑2 rings		0.09	0.06	0.03	0.03	0.08	0.03
ACY	Acenaphthylene	0.02	0	0	0.02	0	0
ACE	Acenaphthene	0	0	0	0	0	0
FL	Fluorene	0.02	0.02	0.02	0.02	0.03	0
PHE	Phenanthrene	0.47	0.07	0.08	0.29	0.18	0.08
ANT	Anthracene	0.02	0	0	0.02	0	0
∑3 rings		0.53	0.09	0.1	0.35	0.21	0.08
FLA	Fluoranthene	0.63	0.1	0.07	0.42	0.18	0.16
PYR	Pyrene	0.37	0.05	0.05	0.27	0.11	0.08
BaA	Benzanthracene	0.04	0.02	0.02	0.06	0.02	0.02
CHR	Chrysene	0.11	0.02	0.02	0.08	0.02	0.04
∑4 rings		1.15	0.19	0.16	0.83	0.33	0.3
BbF	Benzo(b)fluoranthene	0.11	0.04	0.04	0.13	0.06	0.05
BkF	Benzo(k)fluoranthene	0.03	0.01	0	0.03	0.02	0.01
BeP	Benzo(e)pyrene	0.07	0.03	0.04	0.05	0.03	0.03
BaP	Benzo(a)pyrene	0.03	0.01	0.01	0.04	0.02	0.01
DahA	Dibenzo(a,h)anthracene	0.02	0	0	0.02	0	0
∑5 rings		0.26	0.09	0.09	0.27	0.13	0.1
IcdP	Indeno1,2,3CD-pyrene	0.07	0.03	0.02	0.08	0.04	0.02
BghiP	Benzo(g,h,i)perylene	0.08	0.02	0.03	0.05	0.02	0.02
∑6 rings		0.15	0.05	0.05	0.13	0.06	0.04
Total naphthalenes		0.09	0.06	0.03	0.03	0.08	0.03
PAHs without naphthalenes		2.09	0.42	0.4	1.58	0.73	0.52
∑PAHs		2.18	0.48	0.43	1.61	0.81	0.55

**Table 4:** PAH diagnostic ratios.

	GL	DF	BI	DG	KL	HB	
LMW/HMW	0.397	0.455	0.433	0.309	0.558	0.25	<1 pyrogenic >1 petrogenic
FLA/(FLA + PYR)	0.63	0.667	0.583	0.609	0.621	0.667	<0.4 petrogenic 0.4–0.5 fossil fuel combustion. >0.5 grass, wood, coal comb.
FL/(FL + PYR)	0.051	0.286	0.286	0.069	0.214	0.0	<0.5 petrol emission >0.5 diesel emission
BaA/(BaA + CHR)	0.267	0.5	0.5	0.429	0.5	0.333	<0.2 petrogenic 0.2–0.35 coal comb. >0.35 vehicular
BAP/(BAP + BeP)	0.3	0.25	0.2	0.444	0.4	0.25	<0.5 photolysis ~0.5 fresh particles
IcdP/(IcdP + BghiP)	0.467	0.6	0.4	0.615	0.667	0.5	<0.2 petrogenic 0.2–0.5 petroleum comb. >0.5 grass, wood, coal comb.
BAP/BghiP	0.375	0.5	0.333	0.8	1	0.5	<0.6 non-traffic >0.6 traffic
CHR/(CHR + BAP)	0.786	0.667	0.667	0.667	0.5	0.8	0.25 heavy-duty vehicles 0.39 coal comb. in a power plant 0.5 light-duty vehicles 0.76 coal comb. in households

**Table 5:** The BAP-TEQ and ILCR values for the schools.

School	BAP-TEQ (mg kg^−1^)	ILCRing	ILCRinh	ILCRderm	Risk
GL	0.0786	1.37 × 10^−6^	2.7 × 10^−11^	1.50 × 10^−6^	3.08 × 10^−6^
DF	0.0207	0.36 × 10^−6^	0.7 × 10^−11^	0.45 × 10^−6^	0.81 × 10^−6^
BI	0.0187	0.33 × 10^−6^	0.6 × 10^−11^	0.41 × 10^−6^	0.73 × 10^−6^
DG	0.0925	1.61 × 10^−6^	3.1 × 10^−11^	2.01 × 10^−6^	3.62 × 10^−6^
KL	0.0349	0.61 × 10^−6^	1.2 × 10^−11^	0.76 × 10^−6^	1.37 × 10^−6^
HB	0.0209	0.36 × 10^−6^	0.7 × 10^−11^	0.45 × 10^−6^	0.82 × 10^−6^

**Table 6:** EC50 and EC20 values for <1 mm and <0.1 mm dust fractions.

School	<1 mm Fraction		<0.1 mm Fraction	
	EC50	EC20	EC50	EC20
GL	not toxic	not toxic	not toxic	not toxic
DF	69.84%	0.92%	not toxic	40.62%
BI	not toxic	not toxic	14.42%	3.38%
DG	not toxic	77.73%	38.08%	7.49%
KL	not toxic	not toxic	112.22%	1.69%
HB	not toxic	not toxic	not toxic	not toxic

## Data Availability

The raw data supporting the conclusions of this article will be made available by the authors on request.

## Abbreviations

The following abbreviations are used in this manuscript:

IARC	International Agency for Research on Cancer
ILCR	Incremental Lifetime Cancer Risk
TEF	Toxic Equivalency Factor
